# Soil amelioration impacts viral ecology in saline-alkali lands

**DOI:** 10.1093/ismejo/wrag217

**Published:** 2026-08-26

**Authors:** Junxi Liu, Guixiang Zhou, Lin Chen, Yu Xiao, Yakov Kuzyakov, Congzhi Zhang, Ping Huang, Donghao Ma, Jiabao Zhang

**Affiliations:** State Key Laboratory of Soil and Sustainable Agriculture, Institute of Soil Science, Chinese Academy of Sciences, Nanjing 211135, China; University of Chinese Academy of Sciences, Beijing 100049, China; State Key Laboratory of Soil and Sustainable Agriculture, Institute of Soil Science, Chinese Academy of Sciences, Nanjing 211135, China; State Key Laboratory of Soil and Sustainable Agriculture, Institute of Soil Science, Chinese Academy of Sciences, Nanjing 211135, China; State Key Laboratory of Soil and Sustainable Agriculture, Institute of Soil Science, Chinese Academy of Sciences, Nanjing 211135, China; University of Chinese Academy of Sciences, Beijing 100049, China; Department of Soil Science of Temperate Ecosystems, University of Göttingen, Göttingen 37077, Lower Saxony, Germany; Agro-Technology Institute, Peoples Friendship University of Russia (RUDN University), Moscow 117198, Russia; State Key Laboratory of Soil and Sustainable Agriculture, Institute of Soil Science, Chinese Academy of Sciences, Nanjing 211135, China; Key Laboratory of Reservoir Aquatic Environment, Chongqing Institute of Green and Intelligent Technology, Chinese Academy of Sciences, Chongqing 400714, China; State Key Laboratory of Soil and Sustainable Agriculture, Institute of Soil Science, Chinese Academy of Sciences, Nanjing 211135, China; State Key Laboratory of Soil and Sustainable Agriculture, Institute of Soil Science, Chinese Academy of Sciences, Nanjing 211135, China

**Keywords:** saline-alkali land, soil amelioration, virus–host interactions, DNA-SIP, carbon cycling

## Abstract

The continuous expansion of saline-alkali lands under climate change threatens food security and reduces soil carbon stocks. A common mitigation strategy is soil amelioration, which converts degraded soils back into a fertile and arable state. Microbes play critical roles in soil health and recovery. However, the viruses that infect these microbial communities, and their potential impacts during saline-alkali soil restoration, remain largely unknown. Here, we combined total soil metagenomics and viromics to investigate host-linked viral ecology across four major saline-alkali regions in China, each encompassing two soil amelioration statuses: saline-alkali and reclaimed. We found that viral community structure was shaped by both geography and soil amelioration status, with salinity and alkalinity emerging as key environmental factors. Viral populations were sensitive to soil restoration, showing strong amelioration-status endemism with functional adaptations. Virus–host dynamics ranged from reduced temperate viruses to abundance mismatches in those infecting key carbon-cycling microorganisms, including carbohydrate degraders. ^13^C-cellulose DNA-SIP experiments provided further support for this mismatch by tracing assimilated carbon transfer between active host and virus populations. Compared with saline-alkali soils, the relative abundance of hosts in restored soils increased from 39.0% to 61.0%, whereas the linked viruses decreased from 60.6% to 39.4%. Together, these findings reveal an underappreciated role of viruses in shaping saline-alkali soil amelioration trajectories, and could improve management strategies for degraded land recovery and carbon storage.

## Graphical Abstract

**Graphical Abstract:**
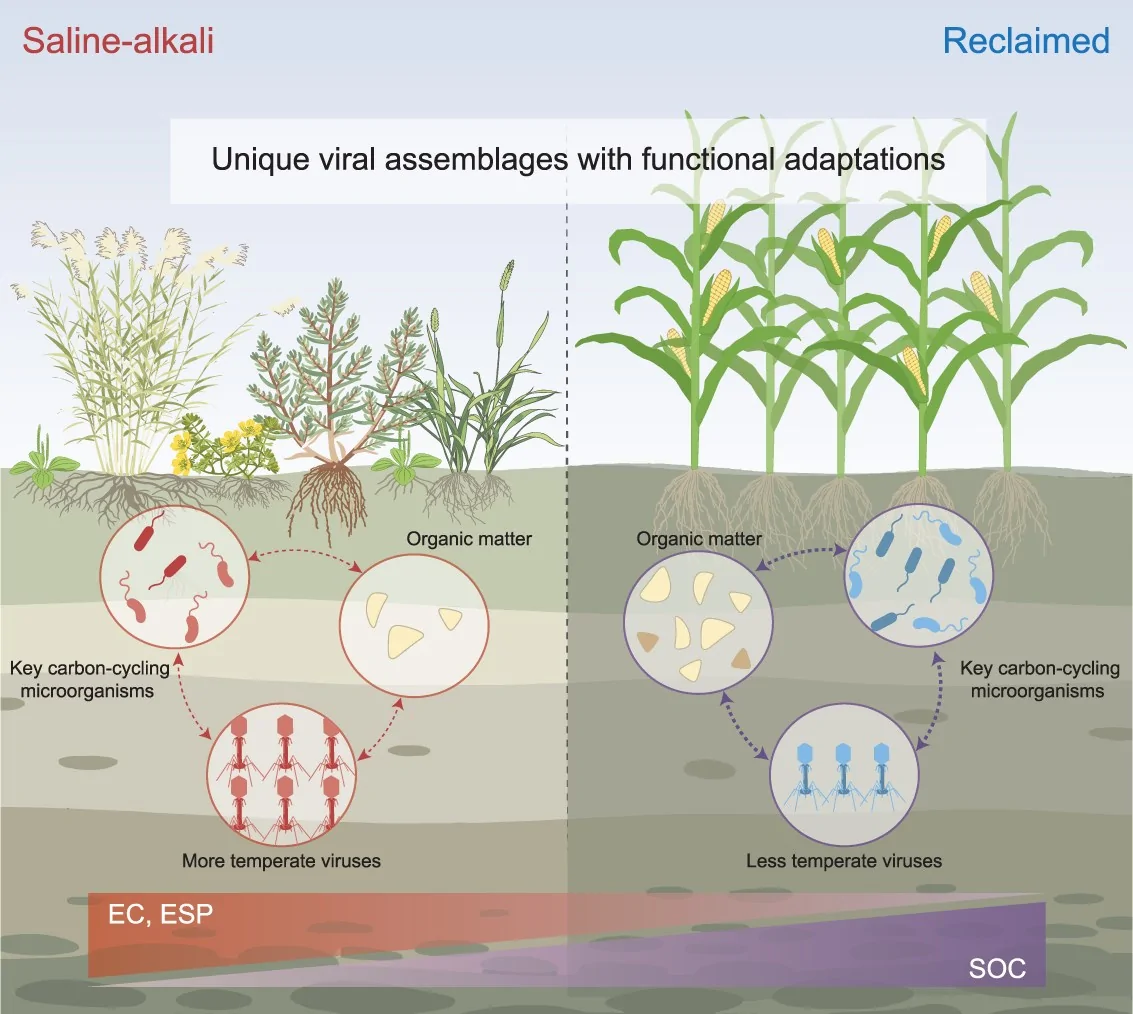

## Introduction

Saline-alkali lands cover ~1 billion ha worldwide and are expanding at a rate of 1–2 million ha per year [1, 2]. Soil salinization has become a severe global issue because it leads to losses of soil organic carbon (SOC), further aggravated by a rapidly changing climate [3]. It is estimated that a one standard deviation increase in salinity of topsoil is associated with SOC declines of ~4.4% in croplands, and 9.3% in natural soils [4]. Therefore, amelioration of saline-alkali lands represents both a critical strategy to address global food security and a sustainable pathway for increasing SOC stocks.

Soil amelioration efforts, often focused on region-specific reclamation techniques such as flood irrigation, drainage systems, and mineralization-driven remediation, aim to alleviate salinity-alkalinity and raise soil carbon storage [5–7]. But the effectiveness of these efforts varies, highlighting the need to identify the key ecological factors of saline-alkali soil recovery. Soil microbiomes play a pivotal role in saline-alkali soil functioning by regulating carbon dynamics and nutrient cycling [8, 9], and successful amelioration likely depends on the reassembly of key microbial processes alongside improvements in physicochemical conditions. Although viruses are increasingly recognized as critical modulators of microbial metabolism and functions with potential biogeochemical consequences [10], their roles in saline-alkali land amelioration remain largely unexplored.

Soil viruses are among the most abundant and diverse biological entities on Earth [11]. By lysing microbial cells and reprogramming host metabolism through auxiliary metabolic genes (AMGs), viruses actively regulate carbon flow and the formation of stabilized carbon pools [12, 13]. These viral effects are highly sensitive to environmental changes [14]. For example, in peatland ecosystems, viral abundance, lifestyles and encoded AMGs shift between damaged and restored sites, highlighting their importance in shaping restoration and carbon cycling [15]. Moreover, conversion of tidal flats into agricultural soils has led to coordinated shifts in virus–nitrifier interaction strategies, thereby influencing nitrification activity [16]. In another pioneering study [17], it was observed that an increase in saline-alkali stress strengthened positive virus–bacteria and virus–fungi correlations in co-occurrence networks. However, little is known about how viruses interact with microbiomes and respond to environmental changes driven by saline-alkali land amelioration.

Host specificity and dependence on host cellular machinery predict that shifts in microbial taxonomic composition and carbon metabolic traits should be accompanied by corresponding changes in associated viral populations and their encoded functions [18]. Amelioration reduces salinity and alkalinity, alleviating microbial stress and altering other soil physicochemical conditions. These environmental changes may create new ecological niches that allow previously rare microbial taxa to establish or increase in abundance, thereby boosting microbial community succession and corresponding shifts in associated viral populations [19]. In parallel, increased resource availability and microbial activity may stimulate the dominance of rapidly growing host populations. According to the “kill-the-winner” hypothesis [20], increased host productivity and density are expected to favor lytic replication and strengthen viral top-down control over dominant microbial taxa, with potential consequences for soil organic matter (SOM) transformation [12].

**Figure 1:**
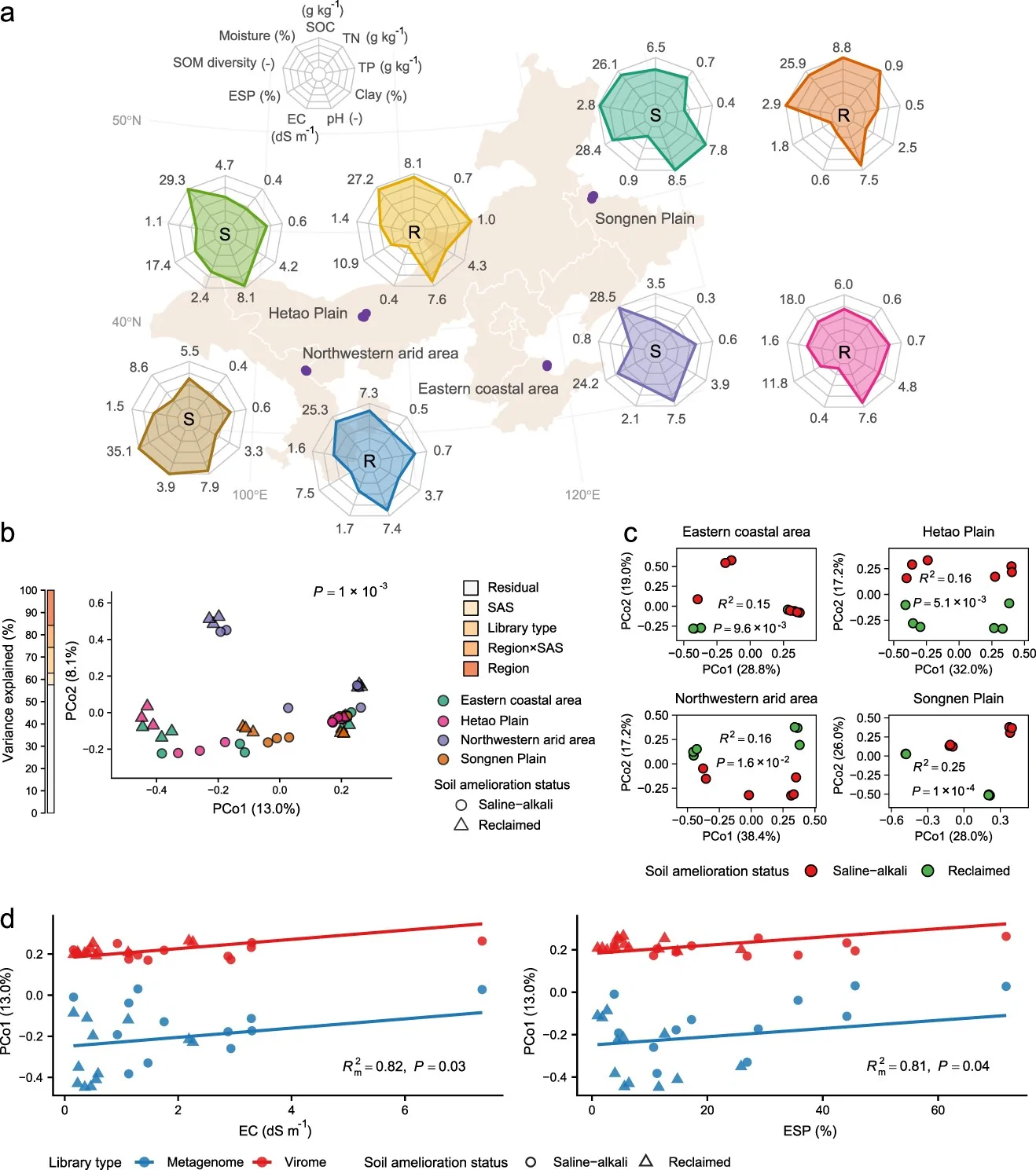
Geography and soil amelioration shape viral community structure. (a) Sampling sites in the four main saline-alkali regions. Radar charts depict key physicochemical parameters of saline and reclaimed soils. S, saline-alkali lands; R, reclaimed lands. (b) PCoA of viral community Bray–Curtis dissimilarities across all samples (metagenome and virome libraries; *n* = 48). The bar on the left denotes the percentage of overall variance explained by the independent variables. The reported *P* value indicates the geographic region effect. SAS, soil amelioration status. (c) Region-specific PCoAs of viral community Bray–Curtis dissimilarities (*n* = 12 libraries per region) with PerMANOVA statistics for separation by SAS; exact *R*^2^ and *P* values are shown on the plots. (d) Linear mixed-effects models of viral PCoA axis 1 against EC and ESP, with the environmental variable and library type fitted as fixed effects and soil sample as a random intercept. Fitted lines for metagenome and virome libraries are shown.

Because saline-alkali soils are reclaimed to safeguard food security, soil health, and increase carbon storage, understanding how viruses influence microbiomes and key carbon-cycling processes is essential to evaluate and improve amelioration outcomes. We collected soil samples from the four main saline-alkali regions in China (Fig. 1a and Supplementary Data 1), covering broad range of soil amelioration statuses (SASs), including saline-alkali and reclaimed (i.e. ameliorated) soils. The reclaimed soils had undergone decades of amelioration and now support the growth of crops such as maize [21].

To comprehensively assess virome-related impacts, we combined total soil metagenomes and virus-enriched soil (viromes) to maximize the recovery of environmental viruses including their dependence on the hosts [22, 23]. We hypothesized that (i) soils under different amelioration statuses would harbor unique viral assemblages; (ii) viral populations would broadly mirror their microbial host taxa, with shifts in host carbon metabolic functions across amelioration states matched by changes in their associated viral communities and encoded functions; and (iii) reduced salinity and alkalinity after amelioration would favor kill-the-winner dynamics, in which a virulent lifestyle is favored, and stronger virus–host abundance associations in shaping SOM composition. This study connects viral dynamics with saline-alkali soil amelioration and offers insights toward nature-based solutions for restoring degraded soils.

## Materials and methods

### Study site and sampling

Soil samples were collected between May and October 2022 from four main saline-alkali regions in China: the Songnen Plain, the Eastern coastal area, the Hetao Plain, and the Northwestern arid area. These regions differ in climate, vegetation, and the causes of soil salinization, and together represent major salt-affected landscapes in China [8, 24]. At each region, we used a paired sampling design and established sampling areas representing two SASs: saline-alkali soils and reclaimed soils, with three replicates sampled per SAS. The plots representing different SASs were located within close proximity, with distances ranging from several meters to several hundred meters. The reclaimed soils were formerly saline-alkali lands that had been converted to cropland through decades of amelioration [21]. Amelioration practices varied by region and included irrigation and drainage engineering, agronomic management, and soil amendments (e.g. desulfurization gypsum) [5, 24]. Region-specific soil reclamation and management practices are detailed in Supplementary Text 1. Soil samples from the 0–30 cm layer were collected using a stainless-steel auger (5 cm diameter). Sample metadata and locations are provided in Supplementary Data 1.

### Soil physicochemical analyses

Soil pH was determined in a 1:2.5 (w/v) soil-to-water suspension with a glass electrode. Electrical conductivity (EC) was measured at 25°C in a 1:5 (w/v) soil-to-water extract using a conductivity meter. Exchangeable sodium percentage (ESP) was calculated from exchangeable sodium (ENa^+^) and cation exchange capacity (CEC). Briefly, ENa^+^ was extracted with 1 M ammonium acetate at pH 7.0 and quantified by atomic absorption spectrophotometry. CEC was determined using the sodium acetate method buffered at pH 8.2. ESP was calculated as (ENa^+^/CEC) × 100 [25]. The molecular composition of SOM was characterized using pyrolysis-gas chromatography/mass spectrometry (py-GC/MS) [26]. Soil samples were pyrolyzed using a CDS Pyroprobe 6150 pyrolyzer (CDS Analytical, Oxford, PA, USA). The pyrolysis products were then transferred to a gas chromatography–mass spectrometry system (Agilent 7890A/5975C; Agilent Technologies, Santa Clara, CA, USA) for compound separation and detection following established protocols. Detected peaks were processed and assigned to putative organic compounds by comparison with mass spectral libraries and published compound information. Compound abundances were expressed as percentages of total peak area, grouped into 10 SOM molecular classes based on likely biochemical origins [27], and filtered to remove compounds with mean relative abundance <0.01% across all samples.

Environmental variables were standardized according to analytical objective: centered and scaled across all samples for among-region comparisons, or within each region for saline-alkali versus reclaimed soil comparisons. This approach distinguished regional environmental gradients from local amelioration effects.

### DNA extraction, library construction, and sequencing

Total community DNA was extracted from homogenized soils using the DNeasy PowerSoil Pro kit (Qiagen, Hilden, Germany) according to the manufacturer’s instructions. For virome preparation, viral particles were recovered from soils using a citrate-based desorption protocol [28]. Briefly, triplicate soil aliquots from each sample were mixed with 10 ml of extraction buffer containing 1% potassium citrate (K_3_C_6_H_5_O_7_), 10% phosphate-buffered saline, and 150 mM magnesium sulfate (MgSO_4_). Viral particles attached to soil colloids were released by sequential mechanical disruption, including vortexing for 1 min, manual shaking for 30 s, and agitation at 400 rpm for 15 min at 4°C. The mixtures were then centrifuged at 4700 × g for 20 min at 4°C to remove soil debris, and the supernatants were carefully transferred to sterile tubes.

The desorption procedure was repeated twice for each soil aliquot to maximize viral particle recovery. Supernatants from the three extraction rounds were pooled and passed through 0.2 μm polyethersulfone membrane filters to remove cells and residual particulates. Viral DNA was subsequently extracted from the filtered viral fraction using the DNeasy PowerSoil Pro kit (Qiagen). Library construction was performed for 24 virome samples and 24 total soil metagenome samples. Virome libraries were constructed using the SRSLY kits (ClaretBio, Santa Cruz, CA), following the manufacturer’s protocol, which included PCR amplification during library preparation. No whole-genome amplification was performed prior to library construction, enabling recovery of both ssDNA and dsDNA viral genomes without significant amplification-related biases. Total soil metagenomic libraries were prepared from community DNA, and all libraries were subjected to paired-end sequencing. Viral sequences were recovered from both total soil metagenomes and viromes. Metagenomic assembly, binning, and annotation strategies are provided in Supplementary Text 2.

### Viromics

Viral sequences were identified from assembled contigs using ViWrap (v.1.3.1) [29] with geNomad (v.1.11.2) [30] as the viral detection module, retaining contigs with an initial minimum length of 2 kb [15]. Viral contigs were required to pass geNomad’s default post-classification filters (viral score ≥ 0.7; for sequences ≤2.5 kb, the presence of at least one viral hallmark gene). Read coverage information from the corresponding samples was used for viral genome binning with vRhyme [31]. Both multi-contig viral bins and unbinned single-contig viral genomes were treated as viral metagenome-assembled genomes (vMAGs). During dereplication, only species-representative vMAGs ≥5 kb were retained for downstream analyses.

To generate a unified viral population catalog, vMAGs recovered from both metagenomes and viromes were combined and dereplicated using dRep (v.3.6.2) [32]. Virus species-level representative genomes were defined at 95% average nucleotide identity and 85% aligned coverage, with dRep parameters (`-ignore_genome_quality -pa 0.8 -comW 0 -conW 0 -strW 0 -N50W 0 -sizeW 1 -centW 0`) optimized for non-cellular genomes so that the largest genome was selected as the representative of each cluster. In total, 36 067 viral species-level clusters (vMAGs) were obtained and used for abundance-based analyses (Supplementary Data 2). Filtered reads from all metagenomic and viromic libraries were then mapped back to the unified representative genome set using Bowtie2 (v.2.5.4) [33]. Abundance, genome coverage and coverage breadth were calculated with CoverM (v.0.7.0) [34]. Viral genomes with ≥50% genome breadth in a sample were considered present. Metagenome- and virome-derived abundance profiles were retained separately by sample and library type for ecological analyses. Host associations for vMAGs were predicted using iPHoP (v.1.4.2) [35] with a custom database that combined the default iPHoP host database and high- or medium-quality host MAGs recovered in this study. Only host predictions with confidence scores ≥90% were retained. Of the 36 067 species-representative vMAGs, hosts were assigned to 2048 (5.7%), including 488 of 1247 metagenome-derived vMAGs (39.1%) and 1560 of 34 820 virome-derived vMAGs (4.5%).

To evaluate the novelty of the recovered viruses, vMAGs from this study were compared with published soil [22, 36, 37] and marine [38] viral genome databases using amino acid identity-based genome clustering. Viral protein-coding genes were predicted and translated, and homologous protein clusters were generated using MMseqs2 (v.17.b804f) [39]. Functional annotation of viral proteins was performed against viral and metabolic databases, including PHROGs (release v4) [40] and KEGG KOfam (February 2026 release) [41]. Putative AMGs were identified using conservative filtering based on functional annotation and genomic context, and only curated AMGs with KEGG KOfam annotations were retained for downstream interpretation [15, 42]. Viral lifestyle classifications provided by ViWrap were used to identify temperate viruses, and normalized temperate virus abundance was calculated relative to total viral abundance in each sample.

### Co-occurrence network analyses

Virus–Host–SOM co-occurrence networks were constructed using matched host genomes, species-representative viral genomes and py-GC/MS-derived SOM compounds [43]. Metagenome- and virome-derived viral abundance matrices were analyzed in parallel. Only features present in at least 50% of the samples were retained. Host, virus and SOM abundance matrices were CLR-transformed separately and then combined. Pairwise associations were calculated using Spearman’s correlation, and edges were retained at |*ρ*| > 0.6 and FDR < 0.05.

Networks were visualized with a Fruchterman–Reingold layout [44]. Nodes represented hosts, viruses or SOM compounds, with SOM nodes colored by compound class; edge color indicated positive or negative correlations and edge linetype indicated library type. Modules were identified using Louvain modularity optimization. Based on within-module connectivity (Zi) and participation coefficient (Pi), nodes were classified as peripherals, connectors, module hubs and network hubs [45]. Viral lifestyles were assigned to network viral genomes as virulent or temperate and summarized by SAS.

### ^13^C-cellulose DNA-SIP experiments

DNA-SIP microcosms were set up to identify active cellulose degraders. Soils from the Songnen Plain were selected for microcosm experiments amended with ^13^C-labeled cellulose, with three replicates established for each treatment. Each microcosm was set up in a 50 ml serum bottle containing 4 g dry-weight equivalent of either saline-alkali soil or reclaimed soil, and the moisture content was adjusted to 60% (w/v). Before substrate addition, the microcosms were moistened and pre-incubated in the dark at 20°C for one week. Subsequently, ^13^C-labeled cellulose (1%, w/w; 97 atom % ^13^C, IsoLife, Wageningen, The Netherlands) was added. Each ^13^C-labeled microcosm was paired with an identical ^12^C-control microcosm amended with the unlabeled cellulose.

Microcosms were incubated at 20°C for 6 weeks [46]. After incubation, DNA was extracted from 0.5 g soil using the DNeasy PowerSoil Pro kit (Qiagen). ^13^C-enriched DNA was separated by CsCl density-gradient ultracentrifugation. DNA from the heavy fractions with buoyant densities of 1.725–1.745 g ml^−1^ was pooled, desalted, and concentrated using Amicon Ultra 0.5 ml filters (EMD Millipore, USA) [47]. The ^12^C-control samples were processed in the same way.

The bacterial 16S rRNA gene V1-V3 region was amplified using barcoded primers. Shotgun metagenomic sequencing was performed on ^13^C-enriched DNA and corresponding ^12^C-control DNA. Viral sequences were recovered from the metagenomes, and the bioinformatic analyses were performed using the same workflow as described for the *in situ* soil samples. Detailed procedures for identifying ^13^C-labeled hosts and viruses are available in Supplementary Text 3.

### Statistical analysis

Data analysis was conducted using R (v.4.4.3). SOM diversity was assessed based on the molecular composition of individual soil samples, and ecological metrics (Shannon Index) were calculated using the R package vegan (v.2.6.2). Environmental variation was examined using principal components analysis (PCA), whereas viral community composition was evaluated using Bray–Curtis dissimilarities after Hellinger transformation and visualized by principal coordinate analysis (PCoA). PerMANOVA was used to test the effects of region, SAS, library type, and their interactions. Associations between viral ordination axes or temperate virus abundance and environmental variables were tested using linear mixed-effects models, with library type included as a fixed effect. Differentially abundant viral genomes and host MAGs between SASs were identified using DESeq2, with multiple testing controlled by Benjamini–Hochberg correction. Detailed methods are provided in Supplementary Text 4.

## Results

### Effects of amelioration on soil physicochemical properties

Given our sampling design, we first measured and compared the physicochemical properties of both saline-alkali and reclaimed soils from each representative region. PCA across regions showed that SAS broadly reflected the composition of environmental parameters (Supplementary Fig. 1a). PCA loadings indicated that diversity of SOM composition (SOM diversity) (0.61), and contents of total phosphorus (TP) (0.60), and total nitrogen (TN) (0.54), as well as soil pH (0.55) were the strongest contributors, with ESP (0.51), SOC content (0.48) and EC (0.41) being also important. Along the main PCA axis, reclaimed soils grouped toward higher content of SOC, TN and TP as well as SOM diversity, whereas saline-alkali soils have always higher pH, EC and ESP. These results underscored the role of SAS in structuring soil physicochemical properties across saline-alkali regions.

Region-specific effects were pronounced because separate PCAs within each region revealed specific dominant environmental factors (Supplementary Fig. 1b). Although SAS grouped samples within regions, the degree of separation indicated the importance of the local environmental context and regional differences in reclamation practices. This is consistent with the fact that these regions span major geographic and climatic zones and vary in their degree of soil amelioration. Nevertheless, compared with saline-alkali soils, reclaimed soils had higher organic matter and nutrient levels, and conversely, lower salinity and alkalinity.

### Geography and soil amelioration structure viral and host communities

Next, we examined factors of viral community composition with PCoA (Fig. 1b). Viral community composition showed a strong biogeographic signature (PerMANOVA, proportion of variation explained (*R*^2^) = 0.16, *P* = .001), and SAS explained 5.3% of the variation. The first principal coordinate axis, which explained 13.0% of the total variation, separated communities by library type (metagenomic vs. viromic libraries). However, the impact of SAS became clearer when regions were analyzed separately after controlling for library type (Fig. 1c). Within regions, viral communities from saline-alkali and reclaimed soils were strongly separated (*P* < .05).

Host community composition also impacted viral community structure (Supplementary Fig. 2, Supplementary Results, and Supplementary Data 6). Using linear mixed-effects models, we tested relationships between viral community composition (PCo1) and soil physicochemical variables. Each physicochemical variable and library type was fitted as a fixed effect, and sample ID was fitted as a random intercept to account for paired metagenome and virome observations from the same soil sample. Viral community PCo1 was positively correlated with both EC (marginal *R*^2^ = 0.82, *P* = .03) and ESP (marginal *R*^2^ = 0.81, *P* = .04) (Fig. 1d). TP content and SOM diversity were also associated with PCo1 (*P* < .05, Supplementary Fig. 3). These results provided further evidence that soil amelioration, as represented by changes in salinity, sodicity, and nutrient status, is an important driver in shaping viral community structure.

### Viruses are rarely shared across saline-alkali regions and amelioration statuses

**Figure 2:**
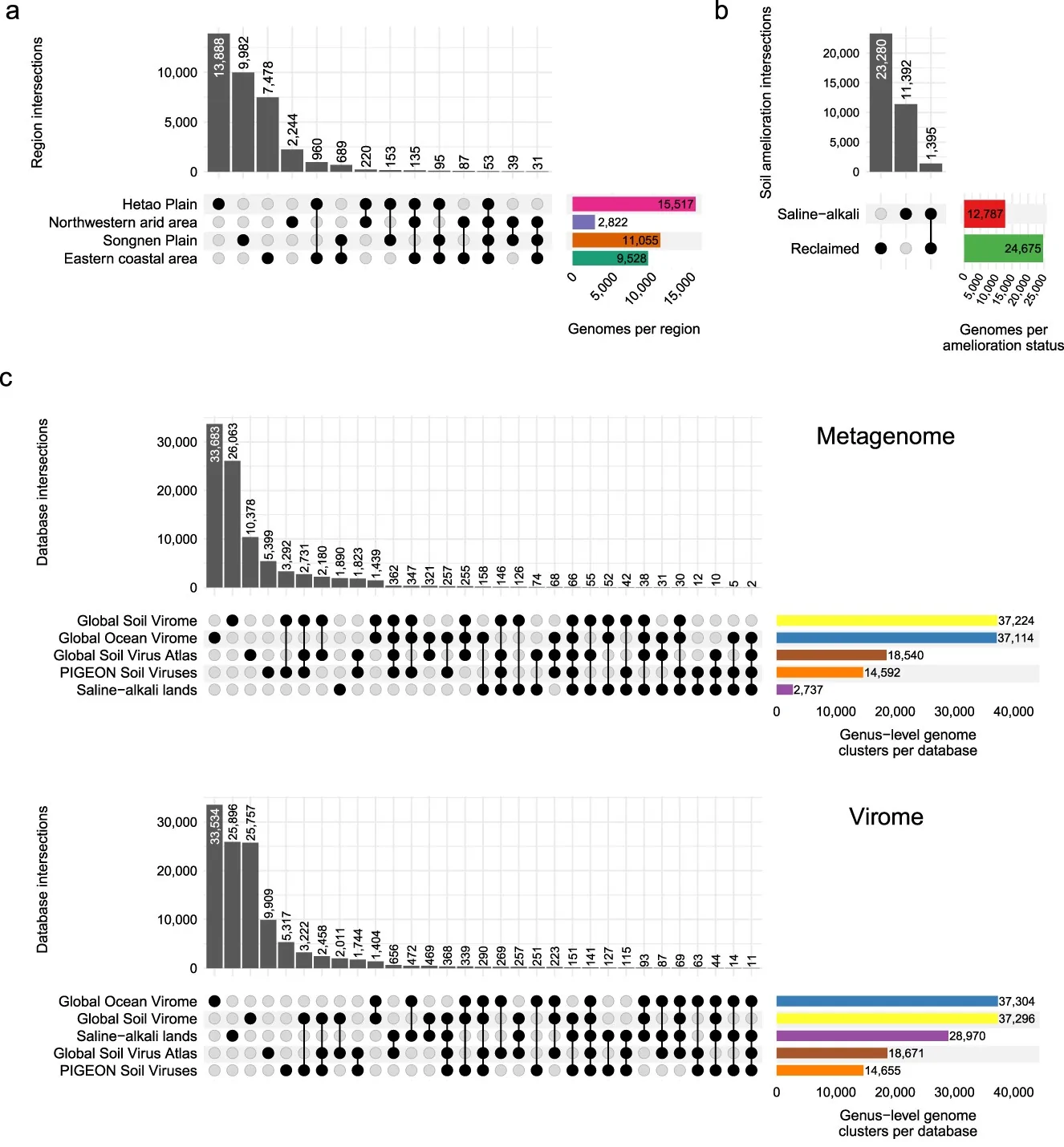
Soil viruses in four main saline-alkali regions are differentially distributed across sample regions, SASs and databases. Detection of species-representative virus genomes across soils from the four regions (a) and soils with different amelioration statuses (b). (c) Intersections of databases represented in genus-level clusters of viruses recovered in this study using metagenomic and viromic approaches and other genomes from three soil virus databases and one marine virus database (*n* = 735 036 genomes). Bars represent clusters with genomes originating from each database in the intersection. Numbers above or inside the bars indicate the total number of genome clusters with genomes originating from each database in the intersection.

Given the strong effects of geography and soil amelioration on structuring viral community across regions, we next examined the degree of endemism among the identified virus species. Most viral populations were restricted to a single region (33 592 of 36 067 vMAGs; 93.1%; Fig. 2a), whereas only 2475 vMAGs (6.9%) were shared among multiple regions. Region-specific viral populations decreased from north to south and from humid to arid regions. Viral population distributions were even more strongly partitioned by SAS (Fig. 2b). Across saline-alkali and reclaimed soils, 34 672 vMAGs (96.1%) were detected in only one soil status, whereas just 1395 vMAGs (3.9%) were shared between the two. Status-specific viral populations were much more numerous in reclaimed soils (23 280 exclusive vMAGs) than in saline-alkali soils (11 392).

To assess whether the viral genomes recovered were largely novel or instead represented in other viral databases, we compared with the three most recent soil virus databases [22, 36, 37] and one marine virus database [38] (Fig. 2c). In the metagenome-derived dataset, 1890 of 2737 genus-level genome clusters (69.1%) were unique to this study, whereas 847 clusters (30.9%) grouped with viruses from at least one virus database. Novelty was even greater in the virome-derived dataset, in which 25 896 of 28 970 clusters (89.4%) were unique to this study, and only 3074 clusters (10.6%) were shared with other databases. Together, these results indicated that soil viruses in four saline-alkali regions contain a large reservoir of locally distinct lineages, alongside a small component of groups shared with other soil and aquatic environments.

### Viral abundance across soil amelioration statuses is inconsistent with dominant hosts

**Figure 3:**
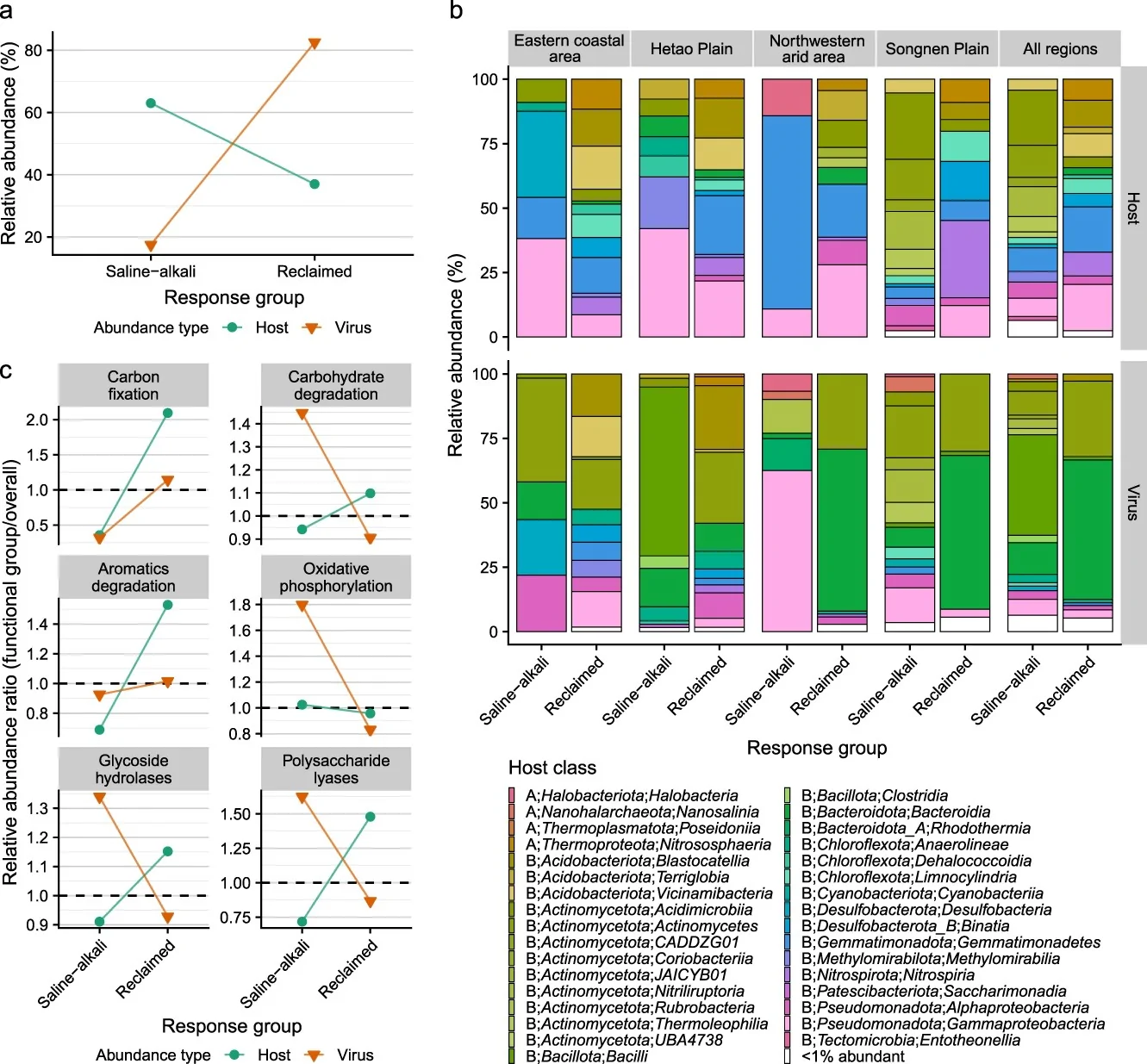
Relative abundance of viruses and hosts across SASs. (a) The relative abundance of viral (*n* = 4181 genomes) and host (*n* = 138 genomes) genomes that are differentially abundant across SAS response groups. (b) Relative abundance of each host class among all differentially abundant host genomes (*n* = 138, top row) and among differentially abundant viral genomes categorized by predicted host class (*n* = 335, bottom row). Host classes are labelled as follows: Domain (A = *Archaea*, B = *Bacteria*); Phylum; Class. Individual classes with <1% relative abundance were grouped into a single “< 1% abundant” category. (c) Abundance ratios of differentially abundant hosts encoding metabolic functions related to key carbon cycling in saline-alkali soils before and after amelioration (*n* = 138 host genomes) and viral genomes predicted to infect hosts carrying these functions (*n* = 58 viral genomes).

Having established that soil amelioration shapes viral communities, we next identified viruses and hosts (Supplementary Data 3) that were differentially abundant between saline-alkali and reclaimed soils. Of the 36 067 species-representative vMAGs, 2048 (5.7%) were assigned to hosts. Using DESeq2, we defined viruses and hosts enriched across SASs as soil amelioration response groups (Supplementary Figs 4 and 5). Across all regions, reclaimed-enriched viruses represented a much larger proportion (85.2%) of differentially abundant viral occurrences than saline-enriched viruses (14.8%) (Fig. 3a). In contrast, hosts showed the opposite trend, with saline-enriched hosts representing 60.2% and reclaimed-enriched hosts accounting for 39.8%.

We next examined the class-level relative abundances of differentially abundant host MAGs and their predicted viruses between saline-alkali and reclaimed response groups (Fig. 3b). Relative abundances of viruses and their hosts varied across regions. When examining all sites together, viruses infecting *Bacillota, Pseudomonadota, Chloroflexota*, and *Desulfobacterota* hosts showed marked decreases in abundance from the saline-alkali to the reclaimed response group. This was accompanied by increases in viruses infecting *Bacteroidota* and *Actinomycetota* hosts, particularly *Bacteroidia* and *Actinomycetes*. These viral abundance shifts did not mirror host changes. For example, whereas the relative abundance of *Gammaproteobacteria* hosts increased from 7.1% in the saline-alkali group to 17.9% in the reclaimed group, the abundance of *Gammaproteobacteria* viruses declined from 6.2% to 3.2%. Similarly, *Bacteroidia* hosts made up only 2.8% of reclaimed-enriched hosts, yet *Bacteroidia*-associated viruses represented 54.1% of reclaimed-enriched viruses. These findings show a clear mismatch between host and viral abundances, indicating that viral responses across SASs cannot be solely explained by host availability and likely also reflect other underlying factors.

### Viral and host dynamics related to key carbon-cycling functions across soil amelioration statuses

Saline-alkali and reclaimed soils have distinct organic matter content and composition. We therefore examined whether viruses infecting hosts with key carbon-cycling functions changed between the two SASs, and whether these changes were consistent with overall viral and host abundance patterns. We calculated the relative abundance of viruses infecting hosts with three carbon metabolic functions (Supplementary Data 4), and normalized to overall viral abundance within the same response group (Fig. 3c). The same calculations were performed for predicted hosts. Because oxidative phosphorylation is a core microbial metabolic process that supports cellular energy generation and may indirectly influence carbon-cycling functions [48], we also included it in the analysis. For carbon fixation, both viral and host abundance increased from saline-alkali to reclaimed soils, with viral abundance rising from 0.32 to 1.14 (+259%, *n* = 10 virus genomes) and host abundance from 0.36 to 2.09 (+486%, *n* = 12 host genomes). Aromatics degradation followed the same direction, with viral abundance increasing from 0.93 to 1.02 (+9.7%, *n* = 63 virus genomes) and host abundance from 0.69 to 1.53 (+122%, *n* = 78 host genomes). Oxidative phosphorylation also tracked in parallel, but here both groups declined—viruses dropping sharply from 1.80 to 0.83 (−54%, *n* = 100 virus genomes), whereas hosts decreased slightly from 1.03 to 0.96 (−6.6%, *n* = 175 host genomes).

Carbohydrate degradation, by contrast, showed an inverse relationship between viral and host responses. Viral abundance fell from saline-alkali (1.45) to reclaimed (0.91) soils (−37%, *n* = 98 virus genomes), whereas host abundance rose from 0.94 to 1.10 (+17%, *n* = 166 host genomes). A similar divergence was observed in CAZy-linked carbohydrate degradation classes. Glycoside hydrolases showed a decline in viral abundance from 1.34 to 0.93 (−31%, *n* = 100 virus genomes) despite an increase in host abundance from 0.91 to 1.15 (+27%, *n* = 179 host genomes). Polysaccharide lyases showed an even stronger decrease in viral abundance from 1.62 to 0.87 (−47%, *n* = 66 virus genomes), whereas host abundance increased from 0.72 to 1.48 (+106%, *n* = 76 host genomes). The more detailed carbohydrate degradation subfunctions further reinforced this host–virus mismatch (Supplementary Fig. 6). Compared to saline-alkali soils, reclaimed soils had decreased viral abundances associated with cellulose degradation, chitin degradation, and hemicellulose debranching, whereas host abundances increased or remained stable. These patterns indicate that although viral and host dynamics were generally coordinated, they differentially responded towards carbohydrate degradation processes, in that the enrichment of functional hosts did not correspond to that of their cognate viruses across SASs.

### Viral proteins are functionally distinct across soil amelioration statuses

**Figure 4:**
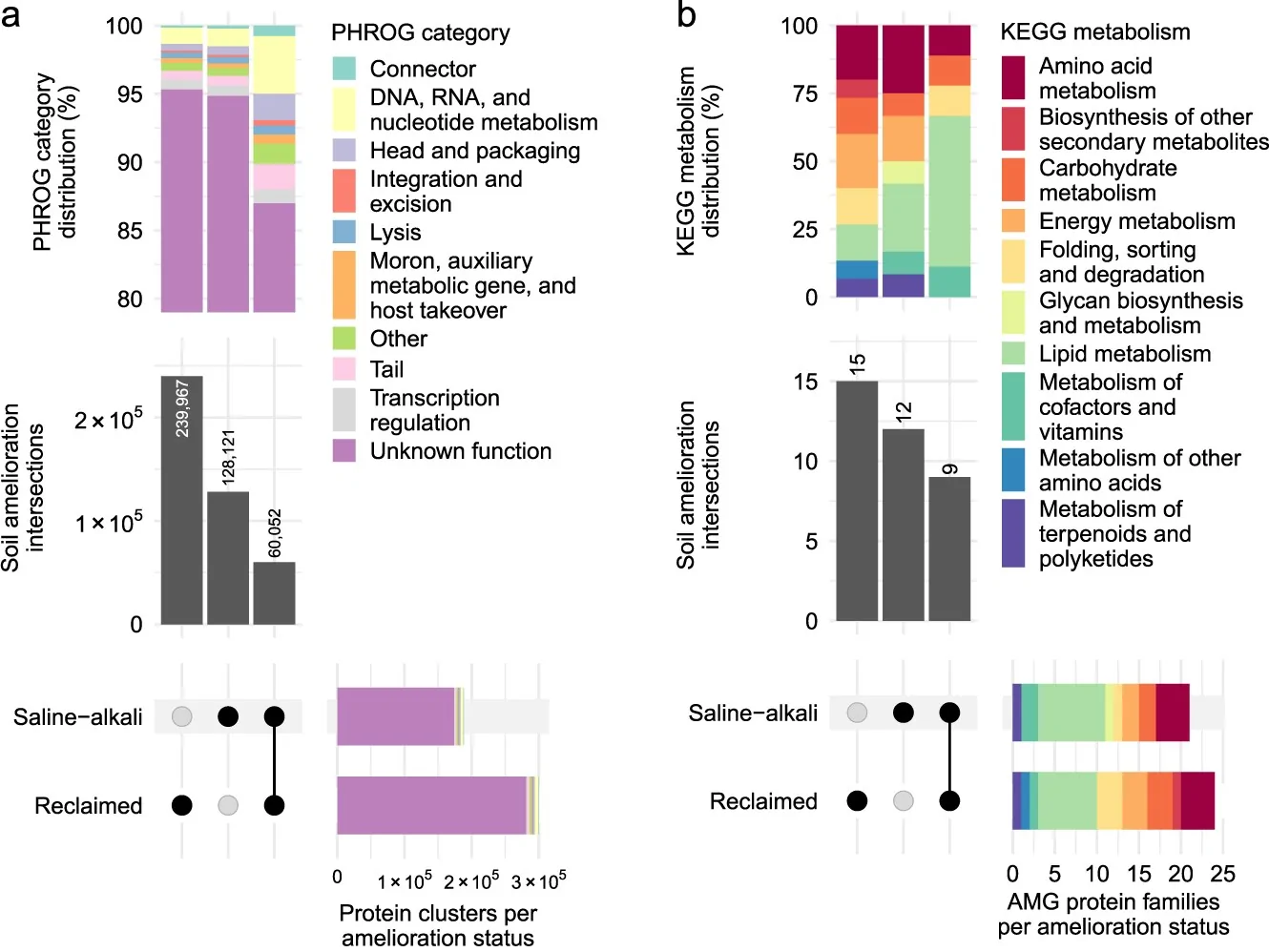
Viral protein-coding genes and AMGs across SASs. (a) UpSet plot showing amino acid identity-based clustering of all viral protein-coding genes (*n* = 428 140 genes). Intersections represent protein clusters with viral proteins from the two SASs, whereas non-intersecting groups represent proteins unique to a single SAS. The distribution of PHROG functional categories across these intersections is shown in the stacked bar plot at the top. (b) UpSet plot of unique KEGG KOfams among viral AMGs (*n* = 100 genes) across SASs. Intersections indicate KOfams shared across different SASs, whereas non-intersecting groups highlight KOfams unique to a single SAS. The stacked bar plot at the top illustrates the distribution of KEGG metabolism categories associated with these viral KOfams across the intersections.

To assess viral functional differences across the two SASs, we clustered protein-coding viral genes from all samples, and examined their distribution between saline-alkali and reclaimed soils (Fig. 4a). The largest protein-cluster intersection was reclaimed-only (239 967 clusters), followed by saline-alkali-only (128 121 clusters). A small number of clusters (60 052) were shared between the two statuses. This pattern indicated that viral protein repertoires are strongly differentiated by SAS, with a substantial fraction of viral functional potential being specific to either saline-alkali or reclaimed soils. At the level of broad functional categories (PHROG database [40]), however, the overall composition remained similar across intersections. The shared protein clusters showed a modestly higher contribution of DNA, RNA and nucleotide metabolism and virion structural categories, such as head and packaging and tail, than the status-specific clusters. No major shift in high-level functional classes was observed. Together, these results suggested that viral proteins were functionally distinct across SASs but retained a conserved broader functional organization.

We next examined viral AMGs and their distribution across the two SASs (Fig. 4b). Similar to the all-protein analysis, AMG protein families were more often status-specific than shared. This result further supported that the metabolic functions encoded by soil viruses differed between reclaimed and saline-alkali soils. Specifically, there was a small increase in the proportion of energy metabolism genes in the reclaimed-only fraction compared with the saline-alkali-only soils, with predicted functions involved in oxidative phosphorylation (K02107 V/A-type H^+^/Na^+^-transporting ATPase subunit G/H). This subtle shift may indicate functional adaptations during soil amelioration, with viruses in reclaimed soils playing a more active role in electron transport-related processes in their hosts for their own fitness benefit [15]. In addition, aromatic and xenobiotic degradation-related functions were status-specific, with benzoate degradation detected only in the reclaimed-only fraction (K03464 muconolactone D-isomerase). Carbohydrate-related AMGs also differed across fractions, with reclaimed-only containing pectate-lyase-associated functions involved in pentose and glucuronate interconversions (K19551 pectate lyase C and K01728 pectate lyase), whereas saline-alkali-only soils were associated with glycolysis/gluconeogenesis (K00121). Overall, viral functions are finely tuned to SASs but remain similar in broader categories.

### Virus–host infection patterns, viral lifestyles, and their associations with SOM composition

**Figure 5:**
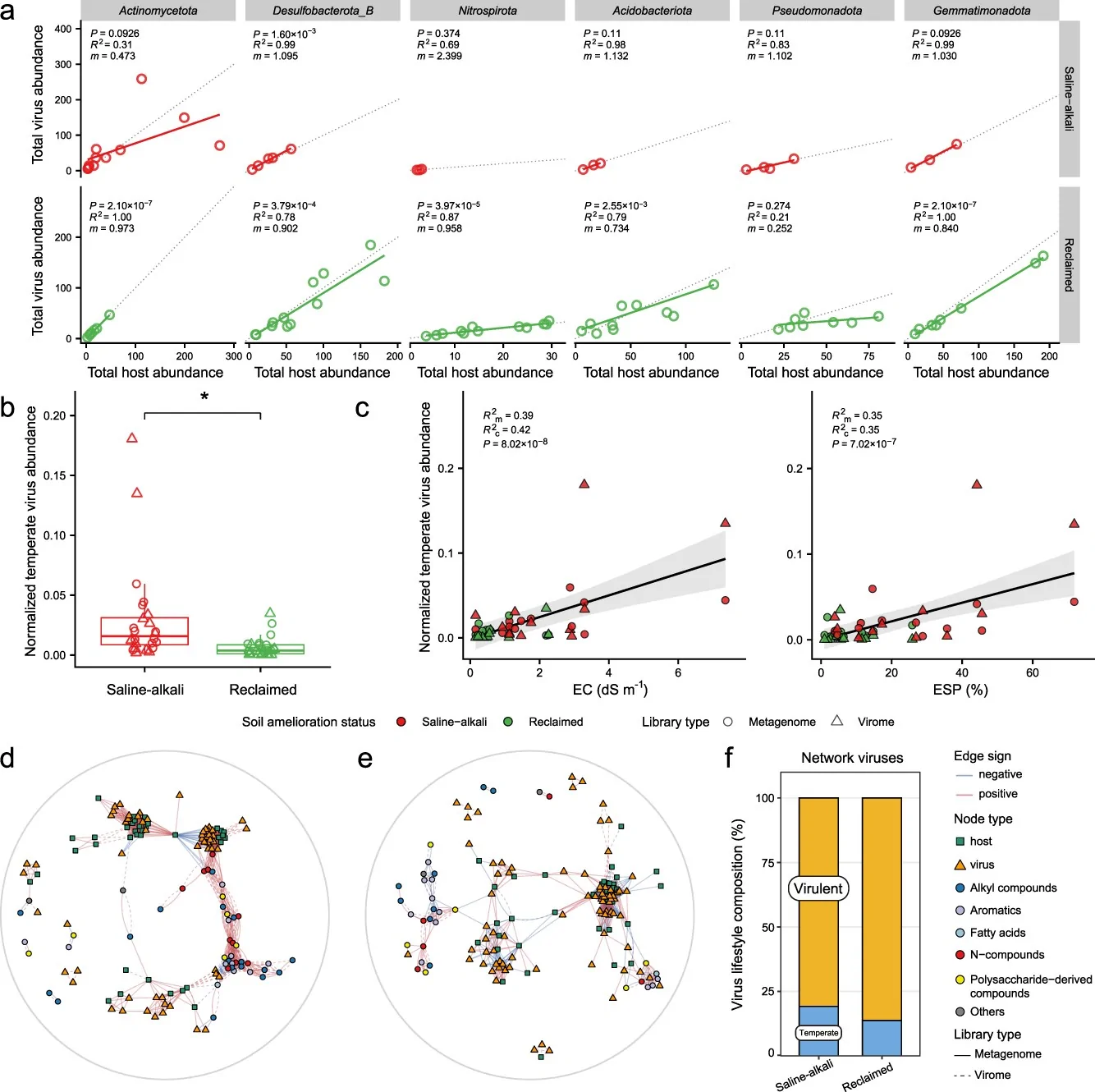
Virus–host interaction patterns across SASs. (a) Linear models of total virus abundance versus total host abundance by SAS. For clarity, only the six host phyla with the highest numbers of observations are shown. Two-sided model statistics are provided for each phylum × SAS combination, where *m* is the slope of the best-fit line (solid line, fitted regression mean) and *P* is the BH-adjusted significance of the host-abundance slope term; dotted lines indicate a hypothetical slope of *m* = 1. (b) Mean normalized abundance of temperate viruses per sample-method library (each temperate viral abundance normalized by total viral abundance in the same library; *n* = 48 libraries from 24 soil samples) across all sites, grouped by SAS. The saline-alkali versus reclaimed contrast was tested using estimated marginal means (two-sided, BH-adjusted) from a linear mixed-effects model with library type as a fixed effect and site as a random intercept. Boxplots: center line, median; box limits, upper and lower quartiles; whiskers, 1.5× interquartile range; points, individual libraries (*P < .05). (c) Linear mixed-effects models predicting normalized temperate virus abundance (as in *B*; *n* = 48 libraries) from EC and ESP, with library type as a fixed effect and site as a random intercept. Black lines show marginal fitted values (population-level mean predictions), and shaded bands show 95% confidence intervals; marginal and conditional *R*^2^ and two-sided Type II ANOVA *P* values are shown on the plots. Networks in saline-alkali (d) and reclaimed (e) soils, in which SOM molecules are represented by circles, microbes by squares, and viruses by triangles. (f) Viral lifestyles in the network.

Viruses depend on their hosts for replication, but the infection activity and lifestyles can shift with soil condition [49]. We therefore examined virus–host infection dynamics using genome abundances from our predicted virus–host pairs (Supplementary Fig. 7). Linear regressions between total virus abundance and total host abundance revealed complex relationships that differed between saline-alkali and reclaimed soils (Fig. 5a). In reclaimed soils, five of the six dominant host phyla showed positive virus–host relationships, e.g. *Nitrospirota* (*R*^2^ = 0.87, BH-adjusted *P* = 4.0 × 10^−5^, *n* = 12 soil samples). Most slopes in reclaimed soils were below 1, suggesting that host genomes generally remained more abundant than their associated viral genomes at the phylum level. By contrast, saline-alkali soils showed a weaker and less consistent pattern. Only *Desulfobacterota_B* had a positive relationship (*R*^2^ = 0.99, BH-adjusted *P* = 1.6 × 10^−3^, *n* = 5 soil samples). The other phyla showed non-significant trends (*P* > .05), often with slopes close to or above 1.

At high host densities, viruses tended to adopt temperate replication strategies, often maintaining infection without causing cell death [20]. To test this assumption, we quantified normalized temperate virus abundance. Temperate virus abundance was higher in saline-alkali soils than in reclaimed soils regardless of whether the four regions were analyzed separately or jointly (BH-adjusted *P* < .05, Fig. 5b and Supplementary Fig. 8). Consistent with this pattern, mixed-effects models showed that normalized temperate virus abundance increased with both EC (marginal *R*^2^ = 0.39, conditional *R*^2^ = 0.42, χ^2^ = 29, *P* = 8.0 × 10^−8^, *n* = 48) and ESP (marginal *R*^2^ = 0.35, conditional *R*^2^ = 0.35, χ^2^ = 25, *P* = 7.0 × 10^−7^, *n* = 48), but decreased with increasing SOC and TN content (*P* < .05, Supplementary Fig. 9), after accounting for region and library type (Fig. 5c). Collectively, these results suggested that soil amelioration decreases the relative abundance of predicted temperate viruses and reshapes virus–host infection dynamics across major host lineages.

To systematically examine how soil amelioration reorganized associations among viruses, hosts, and SOM compounds, we constructed abundance-based co-occurrence networks (Fig. 5d and e). This revealed an increase in the number of network viral nodes with soil amelioration, with distinct interactions across SASs. For example, in saline-alkali soils, aromatics and polysaccharide-derived compounds were mainly embedded in positive links among SOM components, and showed limited connections with viruses and hosts. In reclaimed soils, polysaccharide-derived compounds became more directly coupled to the viral compartment, with the number of virus-associated links doubling compared to pre-amelioration, of which 75% were negative. Globally, network viruses were predominantly virulent in both SASs, but reclaimed soils contained a higher virulent fraction (Fig. 5f). This suggested accelerated virus-mediated microbial turnover and altered SOM transformation following amelioration.

### DNA-SIP experiments on virus–host discordance in cellulose degradation across soil amelioration statuses

**Figure 6:**
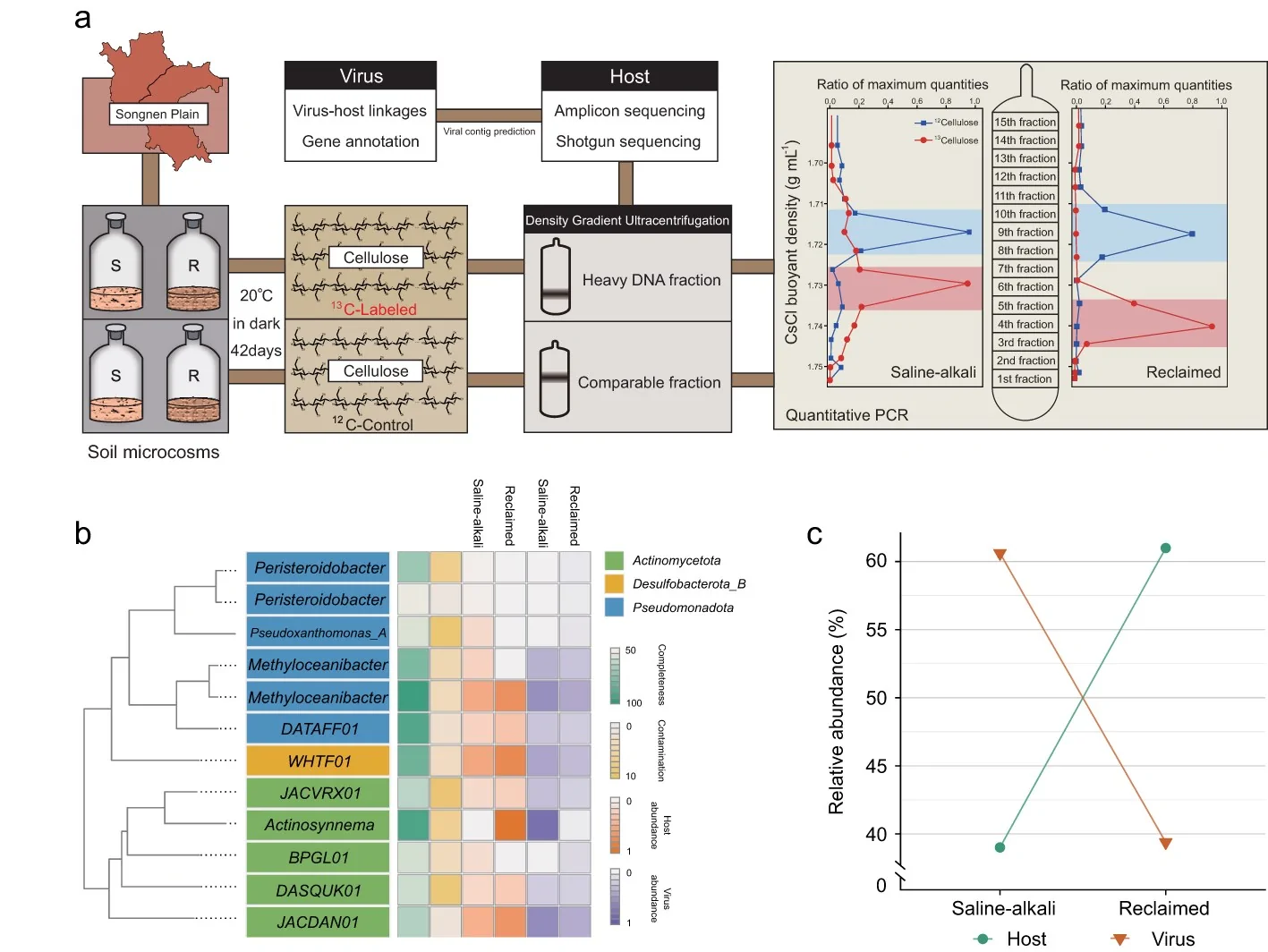
Relative abundance of active cellulose-assimilating hosts and their viruses across SASs. (a) An overview of the samples and methods used in the ^13^C-cellulose DNA-SIP experiments. S, saline-alkali soils; R, reclaimed soils. (b) Phylogenetic tree of the 12 active cellulose-assimilating host MAGs with adjacent heat maps of MAG completeness, contamination, and the normalized abundances of each host MAG and its predicted ^13^C-labeled viral genomes in saline-alkali and reclaimed soils. MAGs are labeled at the genus level and colored by phylum. (c) Active virus–host pairs shown in (b) were differentially abundant across SASs. Relative abundances: hosts, 39.0% in saline-alkali soils vs. 61.0% in reclaimed soils; viruses, 60.6% in saline-alkali soils vs. 39.4% in reclaimed soils (*n* = 6).

To examine the differential abundance patterns between active virus–host pairs involved in carbon degradation across SASs, we performed 6-week microcosm DNA-SIP experiments with saline-alkali and reclaimed soils in Songnen Plain using ^13^C-labeled cellulose and ^12^C cellulose controls (Fig. 6a). The successful targeting of cellulose-assimilating populations was evident in the strong compositional differences between ^13^C and ^12^C amplicon libraries (Supplementary Fig. 10). A total of 33 enriched bacterial classifications spanning 10 phyla were detected in the ^13^C amplicon libraries, enabling the determination of taxa actively degrading cellulose-derived C. More enriched taxa were detected in reclaimed soils (26 OTUs) than in saline-alkali soils (12 OTUs). Differences in predisposition for cellulose catabolism across SASs were evident in the catabolic gene content of metagenome assemblies (Supplementary Fig. 11). However, virus-encoded catabolic genes showed no consistent pattern across SASs (Supplementary Fig. 12).

We further established linkages between 12 active cellulolytic host MAGs and 23 ^13^C-labeled viral genomes using *in silico* prediction (Fig. 6b and Supplementary Data 5). Consistent with the native soil conditions, host relative abundance increased from saline-alkali (39.0%) to reclaimed soils (61.0%), whereas viral relative abundance decreased (60.6%–39.4%) (Fig. 6c). Together, these results provide experimental support for the abundance mismatch between active cellulose-degrading hosts and their associated viruses across SASs.

## Discussion

Saline-alkali soils are among the most widespread degraded land types globally [1, 2]. Soil salinization and alkalinization pose a growing threat to agricultural productivity and ecosystem health worldwide [3]. Amelioration of saline-alkali soils can mitigate this adverse trend by altering salinity stress, SOM content, and nutrient availability, thereby increasing arable land and crop yields [5, 6]. Because carbon cycling in soils is primarily driven by microorganisms [8, 9], understanding how saline-alkali stress and amelioration affect soil microbiomes is crucial for the management of saline-alkali lands and soil fertility improvement. Here, we show that amelioration of saline-alkali soils (i) restructured viral communities alongside biogeographic endemism, (ii) resulted in differential abundance responses between viruses and their hosts across key carbon-cycling functions, (iii) selected for status-specific viral protein repertoires and AMGs, and (iv) reshaped virus–host infection patterns with more virulent interactions coupled to SOM composition, advancing our understanding of how amelioration-induced environmental changes shape soil viral ecology and its contribution to carbon cycling in degraded lands.

We found that viral composition and abundance across SASs often diverged from those of their microbial hosts, rather than tracking host populations. This abundance mismatch was particularly pronounced for viruses linked to carbohydrate degradation, including cellulose and chitin. It was corroborated by DNA-SIP experiments, where active cellulolytic hosts increased from saline-alkali to reclaimed soils, whereas their associated viruses decreased. Such discordance suggests that viral responses are shaped by factors beyond host availability, potentially by salinity and sodicity stress or resource availability shifts. Increased SOM content and other soil constituents may also interfere with viral adhesion to host cells, thereby reducing infection efficiency and the overall number of virions [50]. In parallel, viral proteins showed local adaptation to soil amelioration, with distinct metabolic functions detected in reclaimed soils, including AMGs involved in oxidative phosphorylation and recalcitrant carbon degradation. Although we applied a stringent bioinformatic pipeline for AMG identification [42, 51], further validation of potential viral functions will require controlled microcosm experiments and proteomic analyses [52, 53].

Soil amelioration also altered viral lifestyles, with increased temperate viruses in saline-alkali soils and a transition toward more virulent life strategies in reclaimed soils. Under saline-alkali stress, microbial communities typically show genome streamlining [9] and increased cross-domain cooperation for efficient resource use [8, 17]. Because soil amelioration relieves salinity stress, raises resource availability, and favors the growth of copiotrophic microorganisms [7, 54], putative viral replication strategies could be shifting from “piggyback-the-winner” towards “kill-the-winner” dynamics [20]. However, confirming this interpretation would require direct experimental evidence of changes in infection mode or density-dependent viral control [55].

The higher proportion of predicted virulent viruses in the virus–host–SOM networks of reclaimed soils suggests potentially greater involvement of viruses and their bacterial hosts in shaping SOM chemodiversity [56, 57]. Increased cell lysis caused by viral predation accelerates the mortality of key microbial carbon cyclers, particularly polysaccharide degraders, releasing intracellular contents as labile dissolved organic matter via the viral shunt [12] and contributing to necromass formation [58]. This study used paired viromes and mixed-community metagenomes extracted from the same soil samples, enabling the recovery of most intracellular and extracellular environmental viruses [22, 23, 59]. The proportion of vMAGs assigned to hosts in this study was within the range reported in previous soil virome studies [28, 36, 37]. Nevertheless, advances in long-read sequencing [60], single-cell genomics [61], and high-throughput droplet microfluidics [62] will further improve the reconstruction of complete viral genomes and the quantification of virus–host infection dynamics.

There are several limitations in the current study. First, several analyses rely on relative abundance data, which are inherently compositional and may not reflect changes in absolute viral or host abundance. Second, because the study was based mainly on single-time-point sampling, it cannot directly resolve temporal virus–host dynamics. Third, reclaimed soils differed from saline-alkali soils not only in terms of salinity and alkalinity but also in their long-term agricultural management histories, including cultivation, irrigation, fertilization, and other reclamation practices. Therefore, the observed viral and host responses cannot be attributed exclusively to soil amelioration itself and may partly reflect cumulative management effects. Finally, although co-occurrence networks and genome-based functional annotations provided robust statistical associations and metabolic potential, they do not directly demonstrate changes in SOM transformation rates, decomposition pathways, carbon fluxes, viral activity, or viral gene expression. Future studies combining absolute abundance measurements, time-series sampling, controlled experiments, transcriptomics, and direct measurements of carbon transformation will be required to distinguish predicted functional potential from realized biogeochemical activity.

Because saline-alkali soils are increasingly targeted for amelioration to expand arable land and support food security, increasing our understanding of virus–host interactions is crucial. It will help predict microbial responses and clarify how viral regulation of host populations and metabolism may impact soil carbon accrual and other ecosystem functions. Our findings reveal that viruses do not passively mirror host populations but actively respond to environmental conditions associated with salinity-alkalinity and soil amelioration. These responses yield several measurable signatures that could serve as indicators of amelioration progress: e.g. a decline in temperate virus abundance and a shift in virus–host interactions. Monitoring these viral metrics alongside conventional physicochemical parameters (e.g. EC, ESP, SOC) could provide a more holistic evaluation of soil recovery that complements existing salinity- and fertility-based assessments. Integrating host-linked viral ecology into long-term amelioration monitoring will strengthen our capability to assess and guide saline-alkali land restoration.

## Supplementary Material

Supplementary_material_wrag217

## Data Availability

Metagenomic sequencing data generated in this study have been deposited in the National Center for Biotechnology Information (NCBI) Sequence Read Archive under accession numbers PRJNA1459295, PRJNA1460901, and PRJNA1461145. 16S rRNA gene sequencing reads have been deposited under BioProject PRJNA1461152.
