# Supplementary material for: Soil amelioration impacts viral ecology in saline-alkali lands
Source: ISME J. 2026 Aug 26;20(1):wrag217. doi: 10.1093/ismejo/wrag217 (PMC13584217; doi:10.1093/ismejo/wrag217)
Supplement: Supplementary file 1 [file supplementary_material_wrag217.zip › Supplementary_Information_wrag217.docx]

**Soil amelioration impacts viral ecology in saline-alkali lands**

Junxi Liu^1,2,6^, Guixiang Zhou^1,6^, Lin Chen^1,6^, Yu Xiao^1,2^, Yakov Kuzyakov^3,4^, Congzhi Zhang^1^, Ping Huang^5^, Donghao Ma^1^, Jiabao Zhang^1^*

^1^State Key Laboratory of Soil and Sustainable Agriculture, Institute of Soil Science, Chinese Academy of Sciences, Nanjing 211135, China

^2^University of Chinese Academy of Sciences, Beijing 100049, China

^3^Department of Soil Science of Temperate Ecosystems, University of Göttingen, Göttingen 37077, Germany

^4^Peoples Friendship University of Russia (RUDN University), Moscow 117198, Russia

^5^Key Laboratory of Reservoir Aquatic Environment, Chongqing Institute of Green and Intelligent Technology, Chinese Academy of Sciences, Chongqing 400714, China

^6^These authors contributed equally: Junxi Liu, Guixiang Zhou, and Lin Chen.

*Corresponding author:

Jiabao Zhang, jbzhang@issas.ac.cn

**This file includes:**

Supplementary Methods

Supplementary Results

Supplementary Fig. 1 to 12

Description of Additional Supplementary Files

Supplementary Methods

Text 1 Region-specific reclamation and management practices

The principal reclamation pathways differed among regions. In the Songnen Plain, where saline-sodic soils are widespread, reclamation involved irrigation and drainage management, deep tillage, and the application of Ca-containing amendments (desulfurization gypsum). In the Eastern coastal area, where salinization is associated with shallow saline groundwater and seawater intrusion, reclamation primarily included drainage engineering, freshwater leaching, land leveling, and organic and mineral fertilization. In the Hetao Plain, where irrigation-induced secondary salinization is common, reclamation relied on coordinated irrigation and open-ditch or subsurface drainage, together with deep tillage, straw return, and fertilization. In the Northwestern arid area, characterized by limited precipitation and strong evaporation, reclaimed soils were predominantly managed using water-saving irrigation, including mulched drip irrigation, plastic-film mulching, tillage, and fertilizer application.

The reclaimed soils were primarily planted with maize and wheat, whereas the saline-alkali lands supported naturally occurring vegetation dominated by species such as *Phragmites australis*, *Plantago asiatica*, *Setaria viridis*, *Suaeda salsa*, *Tamarix chinensis*, and *Tribulus terrestris*. To reduce potential confounding effects arising from differences in plant communities, sampling locations were selected to have comparable vegetation cover and similar dominant plant assemblages. In addition, all agricultural sites were managed under a consistent monocropping regime. Vegetation cover did not differ significantly among the sampled plots [1]. The observed responses reflect this integrated amelioration regime and cannot be attributed to any single management component.

**Text 2 Metagenomic assembly, binning and annotation**

Raw metagenomic reads were quality-filtered using illumina-utils (v.2.14) [2] within the anvi'o metagenomic workflow (v.8) [3]. Quality-controlled reads from triplicate samples within each region-by-soil amelioration status combination were co-assembled using MEGAHIT (v.1.2.9) [4] with the `meta-large` preset. Contigs shorter than 1 kb were removed, and assembly quality was assessed using metaQUAST (v.5.3.0) [5]. Filtered reads were mapped back to the corresponding assemblies using Bowtie2 (v.2.5.4) [6] to generate read-recruitment and coverage information for downstream genome binning.

Metagenome-assembled genomes (MAGs) were recovered from co-assemblies using MetaBAT2 (v.2.18) [7], with read-mapping coverage profiles used to improve binning. Binning was performed on contigs ≥ 2.5 kb using default parameters. MAG completeness and contamination were estimated using CheckM2 (v.1.1.0) [8] with the lineage workflow. Bins were classified as high-quality MAGs when completeness was ≥ 90% and contamination was ≤ 10%, and as medium-quality MAGs when completeness was ≥ 50% and contamination was ≤ 10%. A total of 236 medium- and high-quality MAGs were recovered (Supplementary Data 3). Taxonomic classification of medium- and high-quality MAGs was performed using GTDB-Tk (v.2.6.1) [9]. Only medium- and high-quality MAGs were retained as host genomes for virus–host prediction and downstream ecological analyses.

The metabolic potential of host MAGs was annotated using METABOLIC (v.4.0) [10]. We focused on functions relevant to carbon cycling, including carbon fixation, oxidative phosphorylation, carbohydrate degradation and aromatics degradation. Functional assignments were inferred from KEGG module, CAZy module and gene-level annotations reported by METABOLIC (Supplementary Data 4). Hosts were classified as carbon-fixing when at least one of the predefined KEGG modules associated with carbon fixation was detected. Oxidative phosphorylation was considered present when genes or KEGG modules associated with respiratory electron transport and ATP generation were detected. Hosts were classified as aromatic-degrading when at least one of the predefined KEGG modules associated with aromatic compound degradation was present. Because carbohydrate degradation is primarily mediated by individual carbohydrate-active enzymes rather than complete KEGG modules, the "FunctionHit" results from METABOLIC-C were used to determine whether each host MAG encoded carbohydrate degradation functions. These assignments represent predicted genomic metabolic potential rather than direct evidence of metabolic activity.

**Text 3 Identification of ^13^C-assimilating taxa and active cellulolytic host–virus pairs**

To identify bacterial taxa that assimilated ^13^C-labeled cellulose, 16S rRNA gene amplicon data from ^13^C-labeled heavy DNA fractions were compared with the corresponding ^12^C-control fractions. OTUs were screened using a combination of differential abundance analysis (DESeq) and indicator species analysis, following the criteria used in previous studies [11, 12]. An OTU was considered a ^13^C-assimilating taxon if it was selected by at least one of these two methods and showed, on average, at least a threefold higher abundance in ^13^C-labeled libraries than in ^12^C-control counterparts in at least one soil amelioration status.

To identify ^13^C-labeled viral genomes, viral sequences recovered from metagenomes of heavy DNA fractions were compared between ^13^C-cellulose treatments and corresponding ^12^C-cellulose controls. For each viral genome, read coverage or relative abundance in the ^13^C heavy fractions was statistically compared with that in the ^12^C heavy fractions using limma's empirical Bayes moderated *t*-test [13]. Viral genomes were defined as ^13^C-labeled when they were significantly enriched in ^13^C heavy DNA relative to the corresponding ^12^C control after Benjamini-Hochberg false-discovery-rate correction (adjusted *P* < 0.05). This approach allowed us to distinguish viruses associated with active cellulose-derived carbon flow from background viral sequences present in heavy DNA fractions. Only ^13^C-labeled viral genomes that were matched to ^13^C-labeled MAGs through *in silico* host prediction were defined as active cellulolytic host–virus pairs (Supplementary Data 5).

**Text 4 Statistical analysis**

Principal component analysis (PCA) of soil environmental variables was performed using the `prcomp` function in R. For community analyses, viral abundance tables from metagenomes and viromes were aligned to the same unified species-level representative genome catalog, Hellinger-transformed, and converted to Bray-Curtis dissimilarities. Principal coordinate analysis (PCoA) was then used to visualize viral beta-diversity. Differences in viral community composition were tested using PerMANOVA with the adonis2 function in vegan. For the global analysis, library type, region, SAS, and the region-by-SAS interaction were included in the model, with permutations constrained by soil sample identity to account for paired metagenome and virome observations from the same sample. Region-level PerMANOVA models included library type and SAS as explanatory variables. Method-specific treatment separation within each region was further evaluated using ANOSIM.

Associations between viral community structure and soil environmental variables were assessed using linear mixed-effects models. The first PCoA axis of viral community composition was used as the response variable, each environmental variable and library type were fitted as fixed effects, and soil sample ID was included as a random intercept. Marginal *R*^2^ values were calculated to estimate the explanatory power of fixed effects.

Differentially abundant viral genomes were identified using DESeq2 based on mapped read count tables [14]. Viral differential abundance analysis was conducted separately for each region and library type using a likelihood ratio test comparing a full model containing SAS with a reduced intercept-only model. Viral genomes with Benjamini-Hochberg adjusted *P* values < 0.05 were considered SAS-responsive. Differential abundance analysis of host MAGs was performed by region using the same likelihood ratio framework, and host MAGs with adjusted *P* values < 0.05 were retained. For visualization of response patterns, significant viral genomes and host MAGs were transformed into relative abundance, standardized as z scores, and clustered using Ward hierarchical clustering according to their mean abundance patterns across SASs. For comparisons of viral and host response patterns, the metagenome-based and virome-based viral abundance matrices were imported separately, labeled by library type, and subsequently combined to calculate viral relative abundances. Viral response-group assignments were derived from region-specific DESeq2 analyses conducted separately for the metagenome and virome datasets.

Virus-host abundance relationships were evaluated for predicted virus-host pairs. For dominant host phyla, total viral abundance was regressed against total host abundance within each SAS, with library type included as a covariate when both metagenome and virome observations were present. *P* values for slope estimates were adjusted using the Benjamini-Hochberg method. Temperate virus abundance was calculated as the abundance of temperate viruses normalized by total viral abundance in each sample-method observation. Differences in temperate virus abundance between SASs were tested using linear models within each region and linear mixed-effects models across all regions, with library type as a fixed effect and region as a random intercept. Pairwise SAS contrasts were estimated using emmeans and corrected for multiple testing using the Benjamini-Hochberg method. Relationships between normalized temperate virus abundance and environmental variables were further tested using mixed-effects models with environmental variables and library type as fixed effects and region as a random intercept. For virus-host-SOM co-occurrence network analyses, viral abundance matrices derived from metagenome and virome libraries were analyzed separately. Each viral abundance matrix was independently combined with the same host abundance matrix and SOM abundance matrix to construct library type-specific networks.

Supplementary Results

**Independent and shared effects on viral community composition**

To disentangle the independent and shared contributions of host community composition, library type, region, and soil amelioration status, we performed variance partitioning of viral community composition using effect-specific restricted permutations that preserved the paired metagenome–virome design. Variance partitioning showed that these four factors jointly explained 30.7% of viral community variation. The pure fractions were largest for library type (adjusted *R*^2^ = 0.12), followed by host community composition (adjusted *R*^2^ = 0.06), region (adjusted *R*^2^ = 0.02), and soil amelioration status (adjusted *R*^2^ = 0.01; Supplementary Data 6). Shared fractions accounted for a further 9.3%, primarily reflecting variation shared between host community composition and region. Partial db-RDA showed significant pure effects of host community composition (*F* = 1.9, *P* = 0.0001), library type (*F* = 7.8, *P* = 0.0001), region (*F* = 1.5, *P* = 0.0001), and soil amelioration status (*F* = 1.4, *P* = 0.0001). In conclusion, although viral community composition was closely associated with host community structure, region and soil amelioration status also impact viral communities.

Supplementary figures

**Supplementary Fig. 1** Environmental composition of the four saline-alkali land sample sites. (a) Principal components analysis (PCA) of globally scaled environmental parameters for all samples, sites, and soil amelioration statuses (n = 24). The percentages of variance captured by the first two PCA axes are provided. Each point represents an individual soil sample positioned according to its physicochemical parameter values, as summarized by the first two principal components. Ellipses, colored by soil amelioration statuses, represent 95% confidence intervals around the centroid of each group. Arrows indicate the direction and strength of each environmental parameter's contribution to the ordination, with longer arrows signifying stronger influence. (b) PCA for each sampling site, using data scaled within each site to emphasize local variation and minimize broader site-level differences.

**Supplementary Fig. 2** Bacterial and archaeal community composition. (a) Principal coordinates analysis (PCoA) of Bray-Curtis dissimilarities of host metagenome-assembled genome (MAG) community composition across all sample sites. The percentages of variance captured by each PCoA axis are provided. Each point represents a host community of an individual sample. Analysis of similarity (ANOSIM; 999 permutations) shows significant separation by region (*R* = 0.469, *P* = 0.001, unadjusted). (b) PCoA of each sample site with ANOSIM statistics of soil amelioration status.

Supplementary Fig. 3 Linear mixed-effects models of viral PCoA axis 1 against TP and SOM molecular diversity, with the environmental variable and method (library type) fitted as fixed effects and soil sample as a random intercept. Fitted lines for metagenome and virome libraries are shown.

**Supplementary Fig. 4** Average z-scores of DESeq2-significant viral genomes across soil amelioration status. Individual z-scores for each virus genome were averaged in each soil amelioration status (SAS), separately, for each sample site. These trends were used to assign viruses to a particular SAS-responsive group. The two groups within each region represent enrichment in saline-alkali soils or reclaimed soils. Boxplots: center line, median; box limits, upper and lower quartiles; whiskers, 1.5× interquartile range.

**Supplementary Fig. 5** Average z-scores of DESeq2-significant host genomes across soil amelioration status. Individual z-scores for each host genome were averaged in each soil amelioration status (SAS), separately, for each sample site. These trends were used to assign hosts to a particular SAS-responsive group. The two groups within each region represent enrichment in saline-alkali soils or reclaimed soils. Boxplots: center line, median; box limits, upper and lower quartiles; whiskers, 1.5× interquartile range.

**Supplementary Fig. 6** Abundance ratios of differentially abundant hosts encoding key metabolic functions related to carbohydrate degradation in saline-alkali soils before and after amelioration (n = 125 host genomes) and viral genomes predicted to infect hosts carrying these functions (n = 58 viral genomes).

Supplementary Fig. 7 Average virus/host abundance ratios by host family. Virus/host abundance ratios for viruses with predicted hosts, averaged at the family level (rows) across samples and library types containing virus–host pairs (columns). Library type indicates whether each virus–host pair was detected from metagenomic or viromic data. Families are labeled as follows: Domain (A = *Archaea*, B = *Bacteria*); Phylum; Family.

**Supplementary Fig. 8** Abundance of temperate virus genomes across soil amelioration statuses. (a) Mean abundance of all temperate viruses (integrated prophage or containing an integrase/excisionase functional annotation, n = 295 genomes) per metagenome or virome library in each soil amelioration status (SAS) across the four regions (48 libraries from 24 soil samples). Saline-alkali versus Reclaimed contrasts were tested using estimated marginal means (emmeans, two-sided, BH-adjusted): site-specific linear models including library type as a fixed effect, and a linear mixed-effects model with site as a random intercept for the all-sites panel. (b) Mean temperate virus abundance in (a), normalized by total viral abundance in each sample-method library (virulent, temperate and unclassified). Pairwise SAS contrasts were provided. Boxplots: center line, median; box limits, upper and lower quartiles; whiskers, 1.5× interquartile range; points, individual libraries (**P* < 0.05, ***P* < 0.01, BH-adjusted).

**Supplementary Fig. 9** Linear mixed-effects models predicting normalized temperate virus abundance (n = 48 libraries) from SOC and TN content, with library type as a fixed effect and site as a random intercept. Black lines show marginal fitted values (population-level mean predictions), and shaded bands show 95% confidence intervals; marginal and conditional *R*^2^ and two-sided Type II ANOVA *P* values are shown on the plots.

Supplementary Fig. 10 All putative cellulolytic taxa were identified based on differential abundance between ^13^C and ^12^C 16S rRNA gene amplicon sequencing libraries (bacterial OTUs). The lowest possible classifications are given with prefixes corresponding to taxonomic rank. The abundance of each taxon *in situ* (i.e., in unincubated field soil) is represented by scaled circles, with different colors indicating different soil amelioration statuses. For each taxonomic classification (i.e., all OTUs within each taxon), the average abundance ratio between ^13^C and ^12^C-libraries is calculated and displayed across different soil amelioration statuses. A lemniscate (∞) indicates cases where a taxon was detected only in ^13^C-libraries.

**Supplementary Fig. 11** CAZy gene composition in ^13^C- and ^12^C-labeled bacterial metagenomes. The x-axis represents individual metagenomes, and the y-axis represents the most abundant CAZy families. Both axes are clustered based on Bray-Curtis dissimilarity across the full dataset, but only the top 30 CAZy families ranked by reads per million are displayed.

**Supplementary Fig. 12** Viral protein-coding genes labeled with ^13^C and ^12^C across soil amelioration statuses (SASs) in the Songnen Plain samples. (a) UpSet plot of KEGG-annotated viral protein-coding gene clusters across the four SIP treatment groups [saline-alkali (^12^C), saline-alkali (^13^C), reclaimed (^12^C), and reclaimed (^13^C)]; 492 KEGG-mapped entries from 379 representative protein clusters were included. Intersections represent clusters shared across treatment groups, whereas non-intersecting groups represent treatment-specific clusters. The stacked bar plot at the top shows the distribution of KEGG metabolism categories across intersections. (b) UpSet plot of dbCAN-annotated viral protein-coding gene clusters (n = 181), grouped by CAZy class, across the same four SIP treatment groups. Intersections indicate clusters shared across treatment groups, whereas non-intersecting groups indicate treatment-specific clusters. The stacked bar plot at the top shows the distribution of CAZy classes across intersections.

Description of Additional Supplementary Files

**File Name: Supplementary Data 1**

Description: Soil sample metadata and physicochemical parameters.

**File Name: Supplementary Data 2**

Description: Genome-level virus metagenome-assembled genome (vMAG) information. Provided are coassembly metadata, library type, GeNomad viral taxonomy, species cluster assignments, lifestyle prediction, auxiliary metabolic gene (AMG) summaries, and host taxonomy.

**File Name: Supplementary Data 3**

Description: MetaBAT2 bins, CheckM2 quality estimates, and GTDB taxonomic assignments.

**File Name: Supplementary Data 4**

Description: Predicted presence/absence of carbon metabolic functions in host MAGs, inferred from METABOLIC HMMHitNum and dbCAN2Hit results. Functions absent in all MAGs are excluded.

**File Name: Supplementary Data 5**

Description: Active virus-host linkages identified by ^13^C-cellulose DNA-SIP and their abundance profiles.

**File Name: Supplementary Data 6**

Description: Restricted-permutation tests and variance partitioning of viral-community composition.

**References**

1. Zhou G, Chen L, Ma L *et al.* Sodicity thresholds alter biodiversity–multifunctionality relationships through fungal dominance and microbial trait‐based strategies. *Glob Change Biol* 2026;**32**:e70843. <https://doi.org/10.1111/gcb.70843>
2. Eren AM, Vineis JH, Morrison HG *et al*. A filtering method to generate high quality short reads using Illumina paired-end technology. *PLoS One* 2013;**8**:e66643. <https://doi.org/10.1371/journal.pone.0066643>
3. Eren AM, Kiefl E, Shaiber A *et al*. Community-led, integrated, reproducible multi-omics with anvi'o. *Nat Microbiol* 2021;**6**:3–6. <https://doi.org/10.1038/s41564-020-00834-3>
4. Li D, Liu CM, Luo R *et al*. MEGAHIT: an ultra-fast single-node solution for large and complex metagenomics assembly via succinct *de Bruijn* graph. *Bioinformatics* 2015;**31**:1674–6. <https://doi.org/10.1093/bioinformatics/btv033>
5. Mikheenko A, Saveliev V, Gurevich A. MetaQUAST: evaluation of metagenome assemblies. *Bioinformatics* 2016;**32**:1088–90. <https://doi.org/10.1093/bioinformatics/btv697>
6. Langmead B, Salzberg SL. Fast gapped-read alignment with Bowtie 2. *Nat Methods* 2012;**9**:357–9. <https://doi.org/10.1038/nmeth.1923>
7. Kang DD, Li F, Kirton E *et al*. MetaBAT 2: an adaptive binning algorithm for robust and efficient genome reconstruction from metagenome assemblies. *PeerJ* 2019;**7**:e7359. <https://doi.org/10.7717/peerj.7359>
8. Parks DH, Imelfort M, Skennerton CT *et al*. CheckM: assessing the quality of microbial genomes recovered from isolates, single cells, and metagenomes. *Genome Res* 2015;**25**:1043–55. <https://doi.org/10.1101/gr.186072.114>
9. Chaumeil PA, Mussig AJ, Hugenholtz P *et al*. GTDB-Tk v2: memory friendly classification with the genome taxonomy database. *Bioinformatics* 2022;**38**:5315–6. <https://doi.org/10.1093/bioinformatics/btac672>
10. Zhou Z, Tran PQ, Breister AM *et al*. METABOLIC: high-throughput profiling of microbial genomes for functional traits, metabolism, biogeochemistry, and community-scale functional networks. *Microbiome* 2022;**10**:33. <https://doi.org/10.1186/s40168-021-01213-8>
11. Wilhelm RC, Singh R, Eltis LD *et al*. Bacterial contributions to delignification and lignocellulose degradation in forest soils with metagenomic and quantitative stable isotope probing. *ISME J* 2019;**13**:413–29. <https://doi.org/10.1038/s41396-018-0279-6>
12. Li J, Sun W, Cao Y *et al*. Increased temperature enhances microbial-mediated lignin decomposition in river sediment. *Microbiome* 2025;**13**:89. <https://doi.org/10.1186/s40168-025-02076-z>
13. Wang B, Gao P, Zhang P *et al*. Elevated temperature simulating heatwaves restructures active nitrifying communities and associated viruses in tidal flats and agricultural soils. *ISME J* 2026;**20**:wrag037. <https://doi.org/10.1093/ismejo/wrag037>
14. Kosmopoulos JC, Pallier W, Malik AA *et al*. Ecosystem health shapes viral ecology in peatland soils. *Nat Microbiol* 2026;**11**:142–54. <https://doi.org/10.1038/s41564-025-02199-x>
